# Deep Reinforcement Learning for Joint Trajectory Planning, Transmission Scheduling, and Access Control in UAV-Assisted Wireless Sensor Networks

**DOI:** 10.3390/s23104691

**Published:** 2023-05-12

**Authors:** Xiaoling Luo, Che Chen, Chunnian Zeng, Chengtao Li, Jing Xu, Shimin Gong

**Affiliations:** 1 School of Information Engineering, Wuhan University of Technology, Wuhan 430070, China; 2 China Three Gorges Corporation, Wuhan 430010, China; 3 School of Computer Sciences, Minnan Normal University, Zhangzhou 363000, China; 4 School of Intelligent Systems Engineering, Sun Yat-sen University, Shenzhen 518107, China; 5 School of Electronic Information and Communications, Huazhong University of Science and Technology, Wuhan 430074, China

**Keywords:** UAV, multi-agent deep reinforcement learning, trajectory planning, access control

## Abstract

Unmanned aerial vehicles (UAVs) can be used to relay sensing information and computational workloads from ground users (GUs) to a remote base station (RBS) for further processing. In this paper, we employ multiple UAVs to assist with the collection of sensing information in a terrestrial wireless sensor network. All of the information collected by the UAVs can be forwarded to the RBS. We aim to improve the energy efficiency for sensing-data collection and transmission by optimizing UAV trajectory, scheduling, and access-control strategies. Considering a time-slotted frame structure, UAV flight, sensing, and information-forwarding sub-slots are confined to each time slot. This motivates the trade-off study between UAV access-control and trajectory planning. More sensing data in one time slot will take up more UAV buffer space and require a longer transmission time for information forwarding. We solve this problem by a multi-agent deep reinforcement learning approach that takes into consideration a dynamic network environment with uncertain information about the GU spatial distribution and traffic demands. We further devise a hierarchical learning framework with reduced action and state spaces to improve the learning efficiency by exploiting the distributed structure of the UAV-assisted wireless sensor network. Simulation results show that UAV trajectory planning with access control can significantly improve UAV energy efficiency. The hierarchical learning method is more stable in learning and can also achieve higher sensing performance.

## 1. Introduction

Nowadays, with the development of unmanned aerial vehicles (UAVs) and the increasing traffic demand on future wireless networks, UAVs can be integrated into wireless networks and used to build an air–ground integrated wireless sensing network for the Internet of Things (IoT), e.g., [1,2,3]. Traditionally, direct links between ground users (GUs) and a remote base state (RBS) can be unreliable due to channel blockage, GU mobility, and limited energy supply. Thanks to enhanced air-to-ground direct channel conditions and UAVs’ fast mobility, UAVs can play an important role assisting GU data sensing and information forwarding to the RBS. UAVs can be used as aerial access points to enhance service provisioning to the GUs or as relay nodes to assist data transmissions beyond the RBS’s service coverage area [4,5]. For example, by leveraging their flexibility in fast deployment, UAVs can serve as mobile access points for emergency rescue [6,7].

Currently, there are still some limitations to joint control of UAV trajectory and transmission-control strategies due to the complexity of high-dimensional optimization, the lack of centralized coordination, and unknown dynamics of network environments, e.g., [8,9,10]. To exploit the performance gain of UAV-assisted wireless networks, UAV trajectory planning is one of the most beneficial design problems to make use of UAV mobility and reshape the network structure dynamically in favor of data transmission, e.g., [11,12,13,14,15,16,17,18,19,20,21,22]. There are many existing works that focus on the trajectory planning problem in UAV-assisted wireless networks. The GUs’ uplink-data transmission strategy is also a critical design aspect for efficient data collection and transmission in UAV-assisted sensing networks. Due to variations in UAV coverage at different locations, GUs have to be smartly divided among different UAVs as a trade-off between interference and network coverage [23,24]. When some UAVs have a low altitude and are closer to the GUs, the UAV may have a restricted coverage area and only serve a limited number of GUs; however, it will have better channel conditions for the GUs under its coverage. In other cases, when more GUs are covered by the same UAV, the sensing information can be of a large amount and thus take up more of the UAV’s buffer space. This implies more sensing time and higher transmission power for the UAV to forward all information to the RBS. Such a performance trade-off motivates us to optimize the UAVs’ access-control strategy jointly with UAV trajectory planning. It is clear that UAV access control depends on UAV trajectories in each time slot and on the time-varying network environment, including the GUs’ spatial distribution, channel conditions, traffic demands, and energy supply. Most of the existing works in the literature focus on energy and spectrum efficiency in UAV-assisted sensing networks by designing UAV trajectories and effective scheduling strategies [25,26,27,28,29].

In this paper, we focus on the joint optimization of UAV trajectory, transmission-scheduling, and access-control strategies in a wireless powered sensor network. The GUs are low-power sensor devices with limited energy supply, but they can harvest and convert RF signals into energy supply. As the UAVs fly over their trajectories, they not only collect the sensing data from the GUs but also adapt their access-control strategies to balance GU energy harvesting and consumption. This can help sustain the GUs’ sensing activities and prolong the lifetime of the sensor network. In particular, we consider a time-slotted frame structure for the UAVs to sense and report GU sensing information. In each time slot, the UAVs decide the optimal hovering locations and the transmission-scheduling strategy for information forwarding. Given the UAVs’ locations, each GU can upload its sensing data via either low-power backscatter communications or conventional RF communications with a higher transmission rate. The GUs’ mode selection between backscatter and RF communications can be optimized to balance the GUs’ energy consumption and traffic demands. The GUs’ access-control strategy can be further optimized at each UAV to balance the sensing and transmission overhead. Considering the non-convexity and complexity in such a high-dimensional control problem, we first propose the multi-agent DRL approach to jointly adapt UAV trajectory and transmission-scheduling and GU mode-selection and access-control strategies via continuous interactions with the network environment. To improve the multi-agent learning efficiency, we further propose a hierarchical learning framework to decompose the control variables into two parts. Based on the UAVs’ local observations, the UAV trajectory and scheduling strategy is firstly updated by the upper-layer MADDPG algorithm. Then, given the fixed sensing locations, the GU mode-selection and access-control strategies can be further adapted by the lower-layer DQN method. Our simulation results demonstrate that the hierarchical learning framework has more preferable convergence performance and achieves a significantly higher reward than the conventional MADDPG algorithm.

## 2. Related Works

### 2.1. Multi-UAV-Assisted Wireless Networks

Many traditional optimization methods are applied to solve the problems of trajectories, resource allocation, and scheduling in UAV-assisted wireless sensor networks. To jointly optimize UAV trajectory, resource allocation, and power-allocation strategies, a non-convexity and combinatorial problem was formulated in [11], wherein the authors derived an approximate and iterative algorithm to solve it. The authors in [12] aimed to maximize the minimum average data collection rate of all sensing nodes (SNs). However, the problem lacks a closed-form solution for effective power control. Instead, a data regression method was employed to approximate the optimal solution by the block coordinate descent (BCD) method. To maximize the GUs’ sum rate, the author in [13] proposed using an intelligent reflecting surface (IRS) to improve channel conditions. Similarly, the BCD method was used to optimize resource allocation, IRS phase shift, UAV trajectory planning, and transmission power in an iterative manner. Optimization methods typically require complete network information to adapt UAV trajectory-planning and resource-allocation strategies. This becomes inflexible in a dynamic wireless network as the UAVs frequently change their sensing locations. The overhead for information exchange can be extremely high. Additionally, trajectory optimization in the spatial–temporal domain essentially relies on dynamic programming, which is computational demanding in a large-scale UAV-assisted network.

### 2.2. Multi-Agent DRL for UAV-Assisted Wireless Networks

Compared to traditional optimization methods, the recent application of DRL can make the UAVs more adaptive to a dynamic network environment with incomplete information, e.g., imperfect channel conditions and unknown traffic demands. The authors in [14] studied the joint IoTD association, partial offloading, and communication-resource-allocation problem. A multi-agent DDPG algorithm was proposed to maximize the service satisfaction of the IoTD while minimizing its total energy consumption. The authors in [15] proposed an air computing system to provide computing services for ground equipment. Multi-agent proximal policy optimization (MAPPO) was employed to maximize the number of computing tasks within the heterogeneous QoS requirements by jointly optimizing the UAV resource-allocation and task-offloading strategies. The authors in [16] leveraged the twin-delayed deep deterministic policy gradient (TD3) algorithm to plan UAV trajectories and achieve the goal of minimizing task completion delay. The authors in [17] considered complicated spatial- and temporal-coupling in UAV trajectory planning and network formation. A heuristic algorithm was proposed to update the UAVs’ network formation while optimizing UAV trajectories by using the multi-agent deep deterministic policy gradient (MADDPG) algorithm. In particular, each UAV can collect and cache GU sensing data first and then forward the cached data to the next UAV when they meet each other on their trajectories. The authors in [18] proposed a federated multi-agent deep deterministic policy gradient (F-MADDPG) algorithm for UAV trajectory planning to maximize the average spectral efficiency. Federated averaging (FA) is used to eliminate the isolation of data and thus accelerate the convergence of learning. The distributed F-MADDPG (DF-MADDPG) method is further designed to reduce the communication overhead in the distributed architecture. The design idea of a layered learning algorithm appears in many publications. For example, the authors in [19] aimed to minimize the UAV’s total energy consumption. A two-layer hybrid learning algorithm was designed to adapt the UAV’s trajectory by the DRL method in the top layer and then optimize the underlying resource allocation by using a model-based optimization method. The authors in [20] adopted the hierarchical multi-agent DRL (H-MADRL) framework to improve overall energy efficiency in a mobile edge-computing system by jointly optimizing a high-level access point’s beamforming strategy and the low-level users’ offloading decisions. The authors in [21] proposed a hierarchical DRL framework to minimize the age of information in two steps. The first step is to determine the users’ transmission-scheduling strategy through the outer-loop DRL method, and the second step aims to adapt the uplink and downlink transmission strategies of all nodes through an inner-loop optimization method. Different from the above hierarchical learning frameworks, our method in this paper includes two DRL learning layers instead of a hybrid learning and optimization framework. The upper-layer MADDPG is used to solve the UAV trajectory-planning problem, while the lower-layer DQN is used to solve the GU access-control strategy. The authors in [22] studied UAV network formation and trajectory optimization by a hierarchical learning approach. The network formation aims to adapt the UAV-to-UAV links to improve the UAVs’ transmission capabilities. In the outer-loop, a heuristic algorithm is used to adapt the UAVs’ network formation. Given the fixed network formation strategy, UAV trajectory planning is adapted by the multi-agent DDPG algorithm, which is further enhanced by the Bayesian optimization method. Different from [22], our work in this paper assumes that all UAVs are required to report information directly to the base station, and we focused on UAV trajectory planning, GU transmission scheduling, and access control, which were not considered in [22]. Additionally, we design a two-layer learning algorithm in this paper instead of the outer-loop heuristic algorithm, which can be inflexible for a large-scale UAV-assisted wireless network.

### 2.3. UAV-Assisted Sensing Scheduling and Access Control

Given UAVs’ high mobility, it becomes an important task to adaptively update the the GUs’ sensing-scheduling and access-control policies according to the time-varying network environment. The authors in [29] employed UAVs to assist with downlink transmissions in cellular networks. To maximize the users’ sum achievable rate subject to limited fronthaul capacity, mixed-integer nonlinear programming (MINLP) was proposed to jointly design the UAVs’ positions, transmission beamforming, and the UAV-UE association strategies. The authors in [26] proposed a framework for charging scheduling and energy management for UAVs. To maximize charging efficiency, UAVs have to be properly scheduled to fly back to the charging tower. A multi-agent DRL method was developed to achieve collaborative energy sharing between the UAVs and the charging tower. The authors in [27] aimed to collect the latest information from the GUs by minimizing the averaged age-of-information (AoI). A UAV was employed as a relay node to assist with information transmission to the receiver. The authors in [28] used a UAV as an edge cloud that provides data processing services for IoT devices. The goal was to minimize the UAV’s energy consumption while meeting quality-of-service (QoS) requirements. The authors in [25] aimed to navigate a swarm of UAVs to provide optimal communication coverage for mobile users under partial observation. They proposed a stochastic DRL strategy, namely the soft deep recurrent graph network (SDRGN) approach, to reduce the training cost through distributed online learning. Considering the non-convexity and the unavailability of channel state information due to the UAVs’ movement, the deep Q-learning algorithm was used to update the UAVs’ locations, while the difference of convexity algorithm is used to iteratively update the UAVs’ transmission beamforming and UAV-UE association. The authors in [30] considered rate-splitting multiple access (RSMA) to serve multiple GUs simultaneously in a UAV-assisted wireless network, with the goal of maximizing the overall capacity. The authors in [31] also considered RSMA for a multi-UAV-assisted downlink wireless network to maximize the multi-user ergodic sum rate. The authors in [32] considered using a UAV as a flying base station to serve multiple GUs. The GUs’ uplink information transmissions to the UAV followed the non-orthogonal multiple access (NOMA) strategy to improve spectrum efficiency. GUs’ NOMA transmissions were also studied in [33], which considered a multi-UAV-assisted vehicular communication network.

## 3. System Model

**Figure 1:**
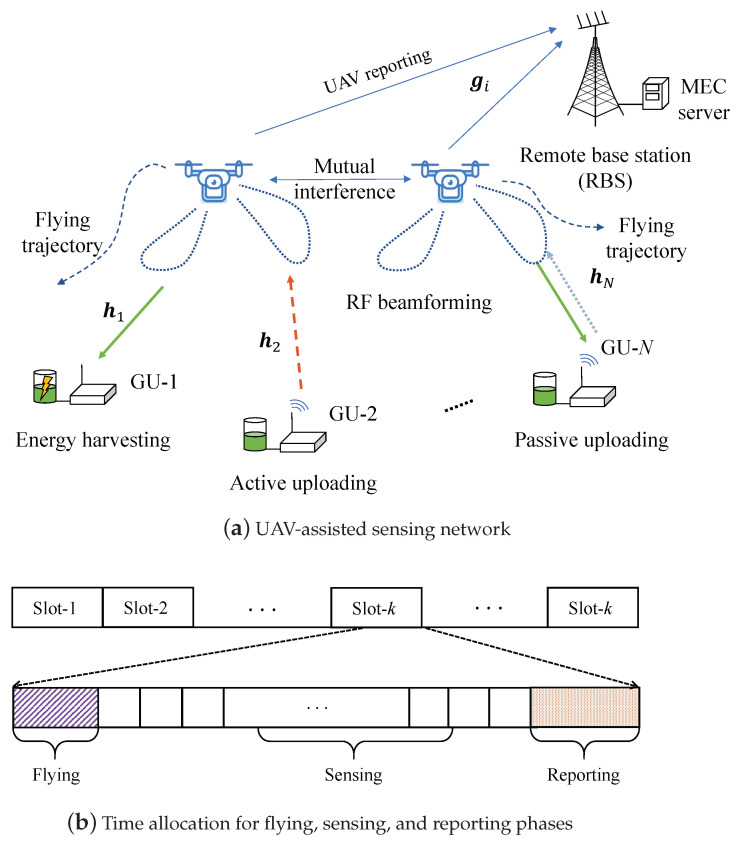
UAV-assisted downlink wireless power transfer and uplink information transmission.

We consider a UAV-assisted wireless network with one RBS and multiple UAVs to serve multiple GUs, as shown in Figure 1a. The set of UAVs is denoted as N={1,2,…,N}, and the set of GUs is denoted as M={1,2,…,M}. Due to blockage or large distances between GUs and the RBS, direct links between GUs and the RBS are unavailable. The UAVs can fly over the GUs, collect the GUs’ sensing data, and then carry the information to the RBS. Each GU can harvest energy from the UAVs’ RF beamforming signals to charge its battery and sustain its operations. Each UAV has *F* antennas, while the GU has a single antenna. Via beamforming optimization, the UAV can control its energy transfer to different GUs and also adapt the uplink transmission rates. Each GU’s sensing data can be uploaded to the UAV by either active RF communications or passive backscatter communications [34], depending on its energy status, the channel condition, and traffic demand. After collecting the GUs’ sensing information, the UAV then forwards the information to the RBS.

### 3.1. UAV Trajectory Planning

UAV trajectory planning is realized in a time-slotted frame structure, as shown in Figure 1b. Each time slot has a fixed length τ, which is further divided into three sub-slots for flying, sensing, and reporting phases. The UAV can fly to a preferable location in the first sub-slot $\tau_{f}$, can collect the GUs’ information during sensing sub-slot $\tau_{s}$, and can then report the information to the RBS in sub-slot $\tau_{d}$. In sensing sub-slot $\tau_{s}$, the UAVs adopt a time-division protocol to collect GU information. In particular, each GU under the UAV’s coverage is allocated a mini-slot $\tau_{z}$. All GUs can upload their information to the UAV one-by-one via active or passive communications. Additionally, each GU can harvest RF energy when the other GUs are actively transmitting. The third sub-slot $\tau_{d}$ is used for the UAV to report its information to the RBS. We assume that the UAV-GU and the UAV-RBS channel conditions are constant in each time slot and may change over different time slots as the UAVs fly their trajectories.

Similar to [17], each UAV-*i*’s trajectory can be defined as a set of locations over different time slots, i.e., ${𝓛}_{i}={\left[\ell_{i}\left(t\right)\right]}_{t\in T}$. Each location is specified by a 3-dimensional (3D) coordinate, i.e, $\ell_{i}\left(t\right)=\left(x_{i}\left(t\right),y_{i}\left(t\right),z_{i}\left(t\right)\right)$. Let $\ell_{0}\left(t\right)$ denote the RBS’s location and $d_{i,0}\left(t\right)$ denote the distance between UAV-*i* and the RBS in slot-*t*. Given that UAV-*i* moves in direction $d_{i}\left(t\right)$ with limited speed $v_{i}\left(t\right)\leq v_{\max}$, UAV-*i*’s location in the next time slot can be updated as $\ell_{i}\left(t+1\right)=\ell_{i}\left(t\right)+v_{i}\left(t\right)\tau_{f}d_{i}\left(t\right)$. We have the following inequalities to regulate UAV mobility:

(1) $$d_{i,j}\left(t\right)≜\left|\right|\ell_{i}\left(t\right)-\ell_{j}\left(t\right)||\geq d_{\min},\;\mathrm{and}\;||\ell_{i}\left(t+1\right)-\ell_{i}\left(t\right)\left|\right|\leq v_{\max}\tau_{f},$$

where $d_{\min}$ denotes the minimum allowable distance between two UAVs to ensure safety.

Given the location $\ell_{m}^{u}$ of GU-*m* on the ground, its distance to UAV-*i* is given by $d_{m,i}\left(t\right)=\left|\right|\ell_{i}\left(t\right)-\ell_{m}^{u}\left|\right|$. We consider a realistic channel model consisting of both line-of-sight (LOS) and non-line-of-sight (NLOS) components. Let $h_{m,i}\left(t\right)\in C^{F\times 1}$ denote the channel vector between UAV-*i* and GU-*m* at the *t*-th slot, which can be modeled as $h_{m,i}\left(t\right)=\sqrt{\psi_{m,i}\left(t\right)}{\tilde{h}}_{m,i}\left(t\right)$, where $\psi_{m,i}\left(t\right)=\omega_{0}{\left(d_{m,i}\left(t\right)\right)}^{-\alpha }$ denotes large-scale fading, while small-scale fading is characterized as follows:

$${\tilde{h}}_{m,i}\left(t\right)=\sqrt{\frac{K}{1+K}}{\bar{h}}_{m,i}\left(t\right)+\sqrt{\frac{1}{1+K}}{\hat{h}}_{m,i}\left(t\right).$$

The first term ${\bar{h}}_{m,i}\left(t\right)$ accounts for the LOS component, and the second term ${\hat{h}}_{m,i}\left(t\right)$ denotes the NLOS component. The Rician factor *K* sets different weights for the LOS and NLOS components. Similarly, we can define $g_{i}\left(t\right)$ as the channel vector from UAV-*i* to the RBS.

### 3.2. GU Access Control and Mode Selection

Given the UAVs’ hovering locations in sensing sub-slot $\tau_{s}$, there may be multiple GUs under the same UAV’s coverage. Some GUs may have worse channel conditions, and thus the data rate for information uploading can be low. This implies that the UAV has to design an access-control strategy to improve the energy efficiency for uplink information transmission. Let $M_{i}\left(t\right)\subset M$ denote the set of all GUs under UAV-*i*’s coverage. Let $M_{i}^{a}\left(t\right)\subset M_{i}\left(t\right)$ denote the set of GUs that are allowed to upload sensing information to UAV-*i*. The other part of the GUs in set $M_{i}\left(t\right)\setminus M_{i}^{a}\left(t\right)$ suspend their data transmission in the current time slot due to insufficient energy or undesirable channel conditions. They can resume data transmission when their channel conditions improve. Let $x_{m,i}\left(t\right)\in \left\{0,1\right\}$ denote the access-control strategy of GU-*m* to UAV-*i* in the *t*-th time slot, i.e., $M_{i}^{a}\left(t\right)=\left\{m\in M_{i}\left(t\right):x_{m,i}\left(t\right)=1\right\}$. We require $\sum_{i=1}^{N}x_{m,i}\left(t\right)\leq 1$ to ensure that GU-*m* only accesses to one UAV in each time slot.

For all GU-*m* in the set $M_{i}^{a}\left(t\right)$, we consider a time division protocol to upload their sensing data. In particular, sensing sub-slot $\tau_{s}$ can be further divided into $|M_{i}^{a}\left(t\right)|$ mini-slots with equal length $\tau_{z}$. Each mini-slot is assigned to one GU in $M_{i}^{a}\left(t\right)$ and can be used for RF active transmission or low-power backscatter communications. For active RF transmission, the received signal at UAV-*i* can be denoted as $\varrho_{m,i}^{a}\left(t\right)=\sqrt{p_{m}}h_{m,i}\left(t\right)\varpi_{m}\left(t\right)+v_{0}$, where $p_{m}$ is GU-*m*’s transmission power, $\varpi_{m}\left(t\right)$ is the information symbol with unit power, and $v_{0}$ denotes the noise signal. Then, the data rate in RF communications is given by

(2) $$r_{m,i}^{a}=\tau_{z}\log_{2}\left(1+p_{m}{\left|h_{m,i}\right|}^{2}\right),$$

where we assume a normalized noise power. In passive data uploading, GU-*m* relies on UAV-*i*’s beamforming signals to backscatter its own information symbols [34]. Let $u_{m,i}\left(t\right)=\sqrt{p_{i}^{A}}w_{m,i}s$ denote UAV-*i*’s beamforming signals in the *t*-th mini-slot, where $w_{m,i}$ denotes the normalized beamforming vector for GU-*m*, $p_{i}^{A}$ denotes the fixed transmission power of UAV-*i*, and *s* is a random symbol with unit power. After GU-*m*’s backscattering, the data rate in passive transmission can be approximated as follows:

$$r_{m,i}^{b}=\tau_{z}\log_{2}\left(1+p_{i}^{A}|\Gamma_{o}{|}^{2}\left|\right|h_{m,i}{\left|\right|}^{2}{\left|h_{m,i}^{H}w_{m,i}\right|}^{2}\right),$$

where $\Gamma_{o}$ is an antenna-specific constant coefficient [35]. For simplicity, we assume that UAV-*i* uses the maximum ratio combining (MRC) scheme when detecting GU-*m*’s information. Hence, we have $w_{m,i}=h_{m,i}/\left|\right|h_{m,i}\left|\right|$, and then we can simplify $r_{m,i}^{b}$ as follows:

(3) $$r_{m,i}^{b}=\tau_{z}\log_{2}\left(1+p_{i}^{A}|\Gamma_{o}{|}^{2}\left|\right|h_{m,i}{\left|\right|}^{4}\right).$$

Similar to [35], we allow each GU to optimally select its transmission mode based on the energy status and channel conditions. Let $z_{m}\left(t\right)\in \left\{0,1\right\}$ denote GU-*m*’s transmission mode selection in the *t*-th time slot, i.e., GU-*m* chooses backscatter communication when $z_{m}\left(t\right)=0$ and switch to RF active communication when $z_{m}\left(t\right)=1$. Let $s_{m,i}\left(t\right)$ denote the size of sensing data uploaded from GU-*m* to UAV-*i* in mini-slot $\tau_{z}$, which can be evaluated as follows:

$$s_{m,i}\left(t\right)=z_{m}\left(t\right)r_{m,i}^{a}\left(t\right)+\left(1-z_{m}\left(t\right)\right)r_{m,i}^{b}\left(t\right).$$

### 3.3. UAV Transmission Scheduling and Buffer Dynamics

In the reporting phase, we use $y_{i}\left(t\right)\in \left\{0,1\right\}$ to indicate whether UAV-*i* is scheduled to forward its data to the RBS. To avoid interference among UAVs, we require $\sum_{i=1}^{N}y_{i}\left(t\right)\leq 1$ to ensure that only one UAV can be scheduled to transmit its data in each time slot. Hence, we expect a dynamic update of each UAV’s data buffer over different time slots. Let $A_{m}\left(t\right)$ denote the size of sensing data arriving at GU-*m* at the beginning of the *t*-th time slot. For each GU-*m*, we assume that $A_{m}\left(t\right)\in \left[A_{m,\min},A_{m,\max}\right]$ is independent and identically distributed (i.i.d) with mean value $\lambda_{m}$. Let $\left(\zeta_{m}\left(t\right),Q_{i}\left(t\right)\right)$ denote the sizes of remaining data in GU-*m*’s and UAV-*i*’s buffers, respectively, which can be updated as follows:

(4) $$\zeta_{m}\left(t+1\right)={\left[\zeta_{m}\left(t\right)-\sum_{i\in N}x_{m,i}\left(t\right)s_{m,i}\left(t\right)+A_{m}\left(t\right)\right]}^{+},$$

(5) $$Q_{i}\left(t+1\right)={\left[Q_{i}\left(t\right)+\sum_{m\in M_{i}^{a}\left(t\right)}s_{m,i}\left(t\right)-y_{i}\left(t\right)O_{i}\left(t\right)\right]}^{+},$$

where ${\left[X\right]}^{+}≜\max\left\{0,X\right\}$, and $O_{i}\left(t\right)$ denotes the size of sensing data forwarded to the RBS when UAV-*i* is allowed to transmit in the *t*-th time slot, i.e., $y_{i}\left(t\right)=1$.

(6) $$O_{i}\left(t\right)=\tau_{d}\log\left(1+p_{i,r}\left(t\right)||g_{i}{\left|\right|}^{2}\right),$$

where $p_{i,r}\left(t\right)$ is UAV-*i*’s transmission power in the *t*-th time slot. It is clear that $O_{i}\left(t\right)$ depends on the distance $d_{i,0}\left(t\right)$ and the channel condition $g_{i}$ between UAV-*i* and the RBS.

## 4. Learning for Energy-Efficiency Maximization

We aim to maximize the energy efficiency of the UAV-assisted sensing network by jointly optimizing UAV trajectory, access-control, and transmission-scheduling strategies, as well as GU mode-selection strategies. The overall energy consumption in each time slot includes UAV operation energy consumption during flying and hovering and UAV RF energy consumption during sensing and reporting. For simplicity, we assume that UAV operation energy consumption $e_{i,o}$ is a constant that depends on the overall length of time of flying and hovering. UAV RF energy consumption in sensing $e_{i,s}\left(t\right)$ depends on UAV signal beamforming in different mini-slots. Given a fixed beamforming power $p_{i}^{A}$, UAV RF energy consumption can be evaluated as follows: $e_{i,s}\left(t\right)=\sum_{m\in M_{i}^{a}\left(t\right)}p_{i}^{A}\tau_{z}\left(1-z_{m}\left(t\right)\right)$, where $\tau_{z}$ is the fixed length of each mini-slot. Note that $e_{i,s}\left(t\right)$ relates to the GUs’ access-control strategy $M_{i}^{a}\left(t\right)$ and model selection ${\left\{z_{m}\right\}}_{m\in M_{i}^{a}\left(t\right)}$ in each time slot. For example, when the GUs have insufficient energy supply and rely on backscatter communications more often, the UAVs have to consume more energy for signal beamforming. When a larger set of GUs are allowed to upload their sensing data, this depletes the GUs’ energy faster, especially for those GUs with the worst channel conditions. UAV RF energy consumption in reporting $e_{i,r}\left(t\right)=y_{i}\left(t\right)p_{i,r}\tau_{d}$ can be simply modeled as a linear function of the data transmission time $\tau_{d}$ and UAV transmission power $p_{i,r}\left(t\right)$ when $y_{i}\left(t\right)=1$.

When GU-*m* is associated with UAV-*i*, its active RF communication relies on the energy harvested from UAV-*i*. Let $E_{m}^{h}\left(t\right)$ denote the energy harvested by GU-*m* in the *t*-th time slot. Considering a linear energy harvesting model, the harvested energy $E_{m}^{h}\left(t\right)$ can be estimated as follows:

(7) $$E_{m}^{h}\left(t\right)=\sum_{n\in M_{i}^{a}\left(t\right),n\neq m}\mu p_{i}^{A}\tau_{z}\left(1-z_{n}\left(t\right)\right)E\left[|h_{m,i}^{H}w_{n,i}{\left(t\right)|}^{2}\right],$$

where μ is the energy conversion efficiency. Note that the energy harvesting model in (7) can be easily extended to a more practical nonlinear model. When some other GU-*n* is backscattering its information to UAV-*i*, i.e., $z_{n}\left(t\right)=0$, GU-*m* can harvest RF power from UAV-*i*’s beamforming signal $u_{n,i}\left(t\right)=\sqrt{p_{i}^{A}}w_{n,i}\left(t\right)s$. Therefore, we have the following energy budget constraint:

(8) $$z_{m}\left(t\right)p_{m}\tau_{z}\leq \min\left\{E_{m}\left(t\right)+E_{m}^{h}\left(t\right),E_{m}^{\max}\right\},$$

where $E_{m}\left(t\right)$ denotes the energy status at the beginning of the *t*-th time slot and $E_{m}^{\max}$ is the maximum battery capacity.

Up to this point, we have defined the energy efficiency Ξ as the time-averaged ratio between the overall throughput received by the RBS and the UAVs’ energy consumption:

(9) $$\Xi ≜\lim_{|T|\to \infty }\frac{1}{\left|T\right|}\sum_{t\in T}\sum_{i\in N}\frac{y_{i}\left(t\right)O_{i}\left(t\right)}{e_{i,o}+e_{i,s}\left(t\right)+e_{i,r}\left(t\right)},$$

which depends on the GUs’ access and transmission-control strategies as well as the UAV trajectory-planning and scheduling strategies. Let $z={\left\{z_{m}\left(t\right)\right\}}_{m\in M,t\in T}$ denote the GUs’ transmission mode-selection strategy. Let $X={\left\{x_{m,i}\left(t\right)\right\}}_{m\in M,i\in N,t\in T}$ denote the GUs’ access-control strategy. Let $𝓛={\left\{{𝓛}_{i}\right\}}_{i\in N}$ and $y={\left\{y_{i}\left(t\right)\right\}}_{i\in N,t\in T}$ denote UAV trajectory-planning and transmission-scheduling strategies, respectively. Therefore, we can formulate the energy efficiency maximization problem as follows:

(10) $$\begin{array}{cc} \max_{z,X,y,𝓛} & \Xi (z,X,y,𝓛)\;s.t.\;(1)–(8). \end{array}$$

For simplicity, we consider a fixed beamforming strategy in Problem (10). Thus, the uplink transmission rate and downlink energy transfer to each GU only depend on the channel conditions. The inequalities in (1) define the UAVs’ feasible trajectory-planning strategies. The equalities in (2) and (3) denote the uplink data rates in different transmission modes. The constraints in (4)–(6) describe the buffer dynamics of both UAVs and GUs. The constraints in (7) and (8) ensure sustainable operation of the sensing network. Practically, UAV operation energy consumption $e_{i,o}$ is much larger than the sensing power $e_{i,s}\left(t\right)$ and the reporting power $e_{i,r}\left(t\right)$, which can be ignored in objective (9).

**Figure 2:**
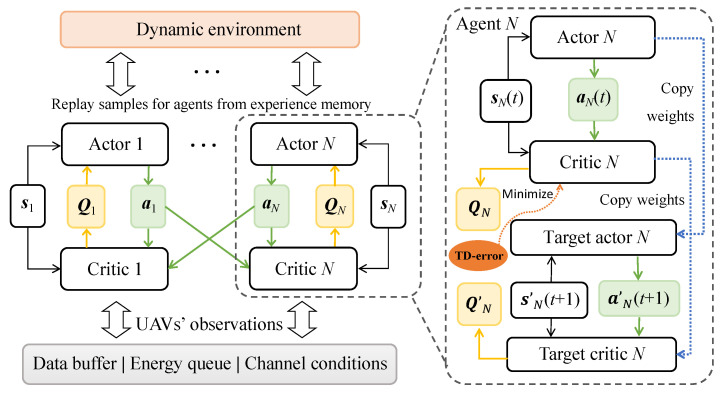
MADDPG framework for UAV centralized training and decentralized execution.

Problem (10) is a combinatorial optimization problem and is difficult to solve optimally. To simplify this problem, we reformulate it into a Markov decision process (MDP), which can adapt the GU access-control and mode-selection strategies as well as the UAV trajectory and scheduling strategies based on continuous interaction with the network environment. Considering that each UAV needs to make decisions independently, we regard each UAV as a decision-making agent and leverage the multi-agent DRL (MADRL) algorithm to solve it. MADRL can effectively coordinate the interactions among multiple agents with large state and action spaces by using a centralized training and decentralized execution scheme [36]. It is built on multiple pairs of actor and critic networks designed for different agents, i.e., the UAVs in this paper, as shown in Figure 2. During the training phase, each critic network needs not only the local observation and action but also the actions of all other agents. This requires information exchange among all UAVs. In online learning, each UAV’s actor network generates its own actions based on local observations, which enables decentralized implementation.

We denote the UAVs’ state in each time slot as $s_{t}=\left(s_{1}\left(t\right),s_{2}\left(t\right),\ldots ,s_{N}\left(t\right)\right)$, which includes all UAVs’ energy storage and data buffers and the channel conditions in the network. Let $\chi_{i}=\left(E_{i},\zeta_{i},Q_{i}\right)$ denote UAV-*i*’s local state. The vector $E_{i}$ represents the energy states of UAV-*i* and all GUs in the set $M_{i}^{a}$ under its coverage. The vector $\zeta_{i}$ denotes the GU buffer states for all GUs in the set $M_{i}^{a}$, and $Q_{i}$ represents UAV-*i*’s buffer state, as shown in (4) and (5), respectively. UAV-*i*’s channel conditions are denoted as $\psi_{i}=\left(h_{i},g_{i}\right)$, where $h_{i}$ denotes the GU-UAV channels for uplink information transmission and $g_{i}$ is the channel vector from UAV-*i* to the RBS. Hence, for each UAV-*i*, its state can be denoted as $s_{i}\left(t\right)≜\left(\chi_{i},\psi_{i}\right)$. We assume that all the states can be measured at the beginning of each sensing slot. The UAVs’ actions in each time slot are defined as the vector $a_{t}=\left(a_{1}\left(t\right),a_{2}\left(t\right),\ldots ,a_{N}\left(t\right)\right)$. Motivated by the optimization problem in (10), each UAV-*i*’s action will include the GUs’ access control $X_{i}={\left[x_{m,i}\left(t\right)\right]}_{m\in M_{i}^{a}}$ and the mode selection $z_{i}={\left[z_{m}\left(t\right)\right]}_{m\in M_{i}^{a}}$, as well as UAV-*i*’s schedule $y_{i}\left(t\right)$ and trajectory $\ell_{i}\left(t\right)$. For simplicity, we denote $a_{i}\left(t\right)≜\left(z_{i},X_{i},y_{i},\ell_{i}\right)$ as the action vector for each UAV.

We denote UAV-*i*’s long-term reward as $R_{i}=\sum_{t=0}^{T}\varsigma^{t}r_{i}\left(t\right)$, where ς∈(0,1) is a discounting factor and $r_{i}\left(t\right)$ denotes the instant reward in each time slot. In our problem, we aim to collect all GU sensing data as much as possible and forward them to the RBS with minimum delay. Hence, the throughput includes two parts, i.e., one part denotes the size of uplink data transmission and the second part denotes the size of data forwarded to the RBS. Additionally, a penalty term $r_{p}\left(t\right)$ can be added to the reward to avoid interference and collision between UAVs. As such, we define each UAV-*i*’s reward as follows:

(11) $$r_{i}\left(t\right)=\frac{\sum_{m\in M_{i}^{a}}x_{m,i}s_{m,i}+\gamma y_{i}O_{i}}{e_{i,o}+e_{i,s}+e_{i,r}}-\eta r_{p}\left(t\right).$$

We omit the time index in (11) for notational convenience. The constant weight parameter γ puts different priority on the throughput in two parts. The penalty term is defined as $r_{p}\left(t\right)=\sum_{j\in N,j\neq i}I\left(d_{i,j}\left(t\right)<d_{\min}\right)$, where I(·) is an indicator function and η can be a large positive value to avoid collision.

After defining the state, action, and reward for each DRL agent, we can proceed to train the actor and critic networks in the MADDPG framework [36], which implements the DDPG algorithm for each agent. Let $\mu_{i}\left(s_{i},a_{i}|\theta_{i}\right)$ denote UAV-*i*’s policy, parameterized by the deep neural network (DNN) (i.e., the actor network) with weight parameter $\theta_{i}$. A delayed copy of the actor network with weight parameter $\theta_{i}^{\prime }$ is also maintained to ensure smooth learning. Similarly, there are also two sets of critic networks with parameters $\omega_{i}$ and $\omega_{i}^{\prime }$ to estimate the Q-values for each state–action pair. Both $\theta_{i}^{\prime }$ and $\omega_{i}^{\prime }$ are delayed copies of $\theta_{i}$ and $\omega_{i}$, respectively. We denote $\theta_{t}=\left(\theta_{1},\theta_{2},\ldots ,\theta_{N}\right)$ and $\omega_{t}=\left(\omega_{1},\omega_{2},\ldots ,\omega_{N}\right)$ as the sets of DNN parameters for all UAV actor and critic networks, respectively. It is clear that each agent-*i*’s expected reward $J_{i}\left(\mu_{i}\right)≜E_{\mu_{i}}\left[R_{i}\right]$ becomes a function of the weight parameters $\left(\theta_{i},\omega_{i}\right)$. By the policy gradient theorem [37], the maximum reward can be evaluated based on the gradient in terms of $\theta_{i}$:

(12) $$\nabla_{\theta_{i}}J\left(\mu_{i}\right)=\nabla_{\theta_{i}}\mu_{i}\left(s_{i},a_{i}|\theta_{i}\right)\nabla_{a_{i}}Q_{i}^{\mu_{i}}\left(s_{t},a_{t}|\omega_{i}\right).$$

Note that agent-*i*’s policy $\mu_{i}\left(s_{i},a_{i}|\theta_{i}\right)$ depends on the local state $s_{i}$, while its Q-value estimation $Q_{i}^{\mu_{i}}\left(s_{t},a_{t}|\omega_{i}\right)$ following the current policy $\mu_{i}$ relates to all UAV actions and states $\left(s_{t},a_{t}\right)$. The update to the critic network’s DNN parameter $\omega_{i}$ also follows a gradient descent approach to minimize the squared error between the Q-value estimation and the one-step look-ahead target Q-value:

(13) $$\min E\left[|Q_{i}^{\mu_{i}}\left(s_{t},a_{t}|\omega_{i}\right)-v_{i}{|}^{2}\right]$$

where the target Q-value is given by $v_{i}=r_{i}\left(t\right)+\gamma Q_{i}^{\mu_{i}^{\prime }}\left(s_{t}^{\prime },a_{t}^{\prime }|\omega_{i}^{\prime }\right)$ and $\mu_{i}^{\prime }$ denotes the target actor network. The complete solution procedure is shown in Algorithm 1. After centralized training, each UAV-*i* can follow its actor network to generate its action $a_{i}=\mu_{i}\left(s_{i}|\theta_{i}\right)+n_{o}$, where $n_{o}$ denotes random noise to trade-off between exploitation and exploration. The action includes all UAVs’ trajectory and scheduling decisions $\left(\ell_{i},y_{i}\right)$ as well as the access-control and mode-selection decisions $\left(X_{i},z_{i}\right)$ for all GUs under its coverage. Then, all GUs follow UAV-*i*’s decisions $\left(X_{i},z_{i}\right)$ to upload their sensing data, as shown in lines 12–16 of Algorithm 1. After sensing, UAV-*i* either forwards its data to the RBS or holds on until the next time slot, depending on scheduling decision $y_{i}$, as shown in lines 17–21 of Algorithm 1. When all UAVs and GUs have updated their actions, the overall reward is evaluated and used to drive the update of actor networks.

The above MADDPG algorithm provides a general solution framework for high-dimensional optimization problems, as shown in (10). However, it is still challenging to deploy in practice due to the requirement for information exchange and large-scale training. The MADDPG algorithm relies on a centralized training and decentralized execution scheme that requires each UAV to report its local system observation to the RBS, including the channel conditions, energy status, and the offloading decisions of the GUs under its coverage. With a large number of UAVs and GUs, the state and action spaces in the MADDPG algorithm will increase drastically. The speed of convergence will slow due to the high-dimensional state and action spaces in a multi-UAV-assisted sensing network. The cost of the information exchange also becomes significant as the number of UAVs increases. Information collection from a large set of UAVs inevitably suffers from excessive delays and slows the training process.
**Algorithm 1** MADDPG for multi-UAV trajectory planning, transmission scheduling, access control, and mode selection1:Initialize all data buffer and energy storage queues2:Initialize channel conditions and UAVs’ observations3:%**UAVs’ trajectory planning**4:**for** each UAV i∈N **do**5:     Collect state information $s_{i}$6:     Generate the action $a_{i}=\mu_{i}\left(s_{i}|\theta_{i}\right)+n_{o}$7:     Update the UAV’s trajectory point $\ell_{i}$8:     Update the UAV’s scheduling decision $y_{i}$9:     Update the GUs’ access-control decision $X_{i}$10:   Update the GUs’ mode-selection decision $z_{i}$11:   Distribute $X_{i}$ and $z_{i}$ to GUs in the set $M_{i}^{a}$12:   %**UAV access control**13:   **for** each GU $m\in M_{i}^{a}$ **do**14:         Transmit in RF mode if $z_{m,i}=1$, otherwise transmit in backscatter mode15:         Update GU-*m*’s data queue and energy states16:   **end for**17:   %**UAVs’ reporting schedule**18:   **if** UAV-*i* is scheduled with $y_{i}=1$ **do**19:         Forward buffered data with size $O_{i}$ to RBS20:         Update the UAV’s data queue21:   **end for**22:   Evaluate the UAV’s reward function $r_{i}\left(t\right)$23:**end for**24:Evaluate all UAVs’ rewards25:Update the target actor and critic networks26:Loop back to step (3)

## 5. A Hierarchical Learning Approach

In this part, we intend to improve the learning efficiency and performance of the conventional MADDPG algorithm by designing a hierarchical framework to reduce the state and action spaces and to avoid frequent information exchange among GUs, UAVs, and RBS. Note that a multi-UAV-assisted wireless network naturally has a hierarchical structure. The RBS is the information receiver and the coordinator of all UAVs. For more efficient sensing, the RBS can deploy and dispatch different UAVs to collaboratively accomplish sensing over a large geographical area. Due to channel fading, the UAVs may have little interference with each other when they are separated in different service regions and aiming to collect sensing information from different sets of GUs. As such, each UAV only cares about its own GUs under its coverage. This implies that the UAV’s local decisions, e.g., the beamforming and scheduling strategy, only affect the local GUs’ access-control and mode-selection strategies. When the UAVs are far apart, they can be viewed as independent devices making their own decisions based on local observations.

### 5.1. Hierarchical Multi-Agent Learning Framework

**Figure 3:**
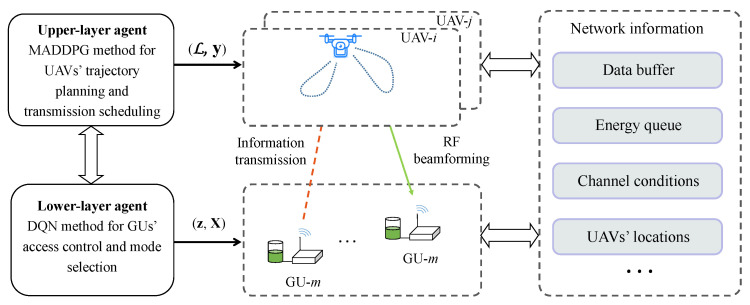
Illustration of the hierarchical framework.

The above observations motivate us to design a hierarchical learning framework to decompose Problem (10) into two sub-problems that can be solved individually and iteratively. The overall learning algorithm includes the upper-layer learning loop for UAV trajectory planning and the lower-layer learning loop for GU access control. Each layer only focuses on a part of the control variables with reduced dimensionality. In particular, we employ the MADDPG algorithm for the UAVs to update their trajectory and transmission-scheduling strategies. Then, given the UAVs’ upper-layer decisions, we further employ the DQN algorithm for each UAV to update the GUs’ access-control strategies under its coverage. As illustrated in Figure 3, each UAV is viewed as an independent agent in the upper-layer learning framework. The centralized training phase of the MADDPG algorithm can be performed by the RBS. After that, each UAV updates its trajectory and beamforming strategy in a distributed manner by individual actor networks based on local observations. When the UAVs move to their new trajectory points, each UAV can collect the status information of the underlying GUs without reporting such information to the RBS. Based on the UAVs’ local observations, each UAV can further decide the GUs’ access-control and mode-selection strategies by the lower-layer DQN method. Then, each GU can follow the UAV’s decision to upload its data to the UAV.

The upper-layer and lower-layer state spaces in each time slot are denoted as $S^{o}$ and $S^{c}$, separately. Correspondingly, the upper-layer and lower-layer action spaces of each UAV are given by $A^{o}$ and $A^{c}$, respectively. The state spaces include the environmental information that can be used to learn the UAVs’ upper-layer and lower-layer actions. The upper-layer state $\chi_{i}^{o}\in S^{o}$ includes each UAV’s channel information $g_{i}$, energy status $E_{i}^{o}$, and buffer size $Q_{i}$ following the dynamics in (5). Thus, we denote it as $\chi_{i}^{o}=\left(g_{i},E_{i}^{o},Q_{i}\right)$. Similarly, the lower-layer state $\chi_{i}^{c}\in S^{c}$ includes all information of the GUs in the set $M_{i}^{a}$ under the UAV’s coverage, including all GUs’ energy statuses $E_{i}^{c}$, the GU-UAV channels $h_{i}$ for uplink information transmission, and the buffer size $\zeta_{i}$ following the dynamics in (4). Thus, we denote it as $\chi_{i}^{c}=\left(h_{i},E_{i}^{c},\zeta_{i}\right)$. The system reward is determined by the state $\left\{S^{o},S^{c}\right\}$ and joint action by $\left\{A^{o},A^{c}\right\}$. As such, we can define the hierarchical learning framework by the information tuple $\left(\left\{S^{o},S^{c}\right\},\left\{A^{o},A^{c}\right\},\left\{R^{o},R^{c}\right\}\right)$. Correspondingly, $\left\{R^{o},R^{c}\right\}$ denotes the reward functions for the upper- and lower-layer agents. In the sequel, we explain the two parts of the algorithm design.

### 5.2. Upper-Layer MADDPG for Trajectory Planning and Scheduling

UAV trajectory-planning and transmission-scheduling strategies can be updated by the MADDPG algorithm according to the UAVs’ local states, including the GUs’ data demands, the UAVs’ energy status, and the channel conditions with the RBS. The RBS can collect all UAVs’ state information and carry out centralized training in the offline phase. For each UAV, the MADDPG algorithm maintains individual actor and critic networks, which have to be trained jointly during the offline phase. After centralized training, the RBS disseminates the actor networks to individual UAVs and allows them to make trajectory-planning and scheduling decisions $A^{o}$ in a distributed manner according to local observations. Then, each UAV hovers at a specific location in the next time slot to collect sensing information from a subset of GUs. The upper-layer action space can be expressed as $A^{o}={\left\{{𝓛}_{i},y_{i}\right\}}_{i\in N}$, which includes the UAV’s trajectory-planning ${𝓛}_{i}$ and transmission-scheduling $y_{i}$ decisions in the next time slot. When the UAV-to-GU distance is less than a threshold, the GU’s uplink signals can be successfully decoded by the UAV, and thus, the GU is considered to be covered by the UAV. It is clear that UAV-*i*’s reward $R_{i}^{o}$ firstly relates to the amount of sensing data received from the GUs under its coverage, which is determined by the lower-layer access-control decision. Let $R_{i}^{c}$ denote the lower-layer sensing reward, which characterizes the amount of sensing data and the resource consumption during the UAV’s sensing phase. The detailed expression of $R_{i}^{c}$ is defined in the next subsection. Secondly, UAV-*i*’s reward $R_{i}^{o}$ also includes the transmission reward, which depends on the UAV’s buffer size and the distance to the RBS. A positive reward is accrued $y_{i}O_{i}$ when UAV-*i* is scheduled to forward its buffered information to the RBS. Additionally, UAV-*i*’s reward $R_{i}^{o}$ has to punish any potential collision with the other UAVs to ensure safety. Similar to (11) for the conventional MADDPG algorithm, the penalty term is defined as $r_{p}\left(t\right)=\sum_{j\in N,j\neq i}I\left(d_{i,j}\left(t\right)<d_{\min}\right)$. As such, we can define the UAV’s reward in the upper-layer learning framework as follows:

(14) $$\begin{array}{c} R_{i}^{o}=R_{i}^{c}+y_{i}O_{i}-r_{p}. \end{array}$$

Let $\pi_{i}^{o}$ denote UAV-*i*’s trajectory-planning and transmission-scheduling policy and $\pi_{-i}^{o}$ denote the other UAVs’ joint policies in the upper-layer learning phase. The long-term expected reward of all UAVs in the upper-layer learning for trajectory and scheduling strategies can be defined as follows:

(15) $$\begin{array}{c} V^{o}\left(\pi_{i}^{o},\pi_{-i}^{o}\right)=E\left[\sum_{t=0}^{\infty }\gamma^{t}R_{i}^{o}\left(s_{t}^{o},a_{t}^{o}\right)\right], \end{array}$$

where the UAVs’ joint states and actions are denoted as $s_{t}^{o}=\left[\chi_{1}^{o}\left(t\right),\chi_{2}^{o}\left(t\right),...,\chi_{N}^{o}\left(t\right)\right]$, and $a_{t}^{o}=\left[a_{1}^{o}\left(t\right),a_{2}^{o}\left(t\right),...,a_{N}^{o}\left(t\right)\right]$, respectively. Each UAV’s policy $\pi_{i}^{o}$ determines its own action given different state, i.e., $a_{i}^{o}\left(t\right)=\pi_{i}\left(\chi_{i}^{o}\left(t\right)\right)$. However, its Q-value estimation $Q_{i}^{\pi_{i}^{o},\pi_{-i}^{o}}\left(s_{t}^{o},a_{t}^{o}\right)$ has to be trained in a centralized manner by the MADDPG algorithm deployed in the RBS, relying on the information collection from all UAVs. By jointly adapting all UAV actions, the value function in (15) can be improved gradually and stabilizes at the convergence. During online execution, each UAV-*i* follows its own policy $\pi_{i}^{o}$ to generate localized trajectory-planning and transmission-scheduling action $a_{i}^{o}\left(t\right)=\left(L_{i}\left(t\right),y_{i}\left(t\right)\right)$ based on UAV-*i*’s local observation $\chi_{i}^{o}\left(t\right)$.

### 5.3. Lower-Layer DQN for GU Access Control and Mode Selection

Given the upper-layer’s trajectory-planning decisions, each UAV is given a new hovering position for information sensing in the next time slot. Thus, in the next step, each UAV updates the access-control decision for the GUs under its coverage. UAV-*i*’s lower-layer action can be defined as $a_{i}^{c}\left(t\right)=\left(z_{i}\left(t\right),X_{i}\left(t\right)\right)\in A^{c}$, including each GU’s access-control and mode-selection decisions in the next time slot. Considering the combinatorial nature of the discrete action space $A^{c}$, we can resort to the classic DQN method to update each UAV’s lower-layer action $a_{i}^{c}\left(t\right)$. According to the local information regarding the GUs, each UAV can adapt its access-control and mode-selection strategy for uplink data transmission to improve the total reward perceived by the UAV. To improve the sensing efficiency, we can define the UAV’s instant reward $R_{i}^{c}\left(t\right)$ in the lower-layer learning framework as a weighted combination of the sensing throughput and the energy consumption as follows:

(16) $$\begin{array}{c} R_{i}^{c}\left(t\right)=\sum_{m\in M_{i}^{a}}x_{m,i}s_{m,i}-\eta_{1}E_{m}^{h}, \end{array}$$

where $\eta_{1}$ is the trade-off parameter for the sensing throughput and the energy consumption. Thus, UAV-*i*’s long-term reward in the lower-layer learning can be denoted as follows:

$$V_{i}^{c}\left(\pi_{i}^{c}\right)=E\left[\sum_{t=0}^{\infty }\gamma^{t}R_{i}^{c}\left(\chi_{i}^{c}\left(t\right),a_{i}^{c}\left(t\right)\right)\right],$$

which only relates to UAV-*i*’s local observations $\chi_{i}^{c}=\left(h_{i},E_{i}^{c},\zeta_{i}\right)$ due to the spatial separation of different UAVs. Therefore, each UAV can individually adapt the GU access-control and mode-selection policy $\pi_{i}^{c}$ to improve and stabilize the value function $V_{i}^{c}\left(\pi_{i}^{c}\right)$.

Given any state–action pair $\left(s_{t}^{c},a_{t}^{c}\right)$, the DQN method deployed in each UAV-*i* estimates its value function $V_{i}^{c}\left(\pi_{i}^{c}\right)$ or the variant Q-function $Q_{i}^{c}\left(\chi_{i}^{c}\left(t\right),a_{i}^{c}\left(t\right)|\omega_{t}\right)$ by two sets of DNNs with weight parameters $\omega_{t}$ and $\omega_{t}^{\prime }$, respectively. To stabilize the learning, the weight parameter $\omega_{t}^{\prime }$ of the target Q network is copied from the online Q network $\omega_{t}$ regularly every few steps. Hence, the target Q-value can be estimated as follows:

(17) $$\begin{array}{c} y_{i}^{c}\left(t\right)=R_{i}^{c}\left(t\right)+\gamma Q_{i}^{c}\left(\chi_{i}^{c}\left(t+1\right),a_{i}^{c}\left(t+1\right)|\omega_{t}^{\prime }\right), \end{array}$$

where the new action $a_{i}^{c}\left(t+1\right)$ is obtained from the online Q network with the parameter $\omega_{t}$ given the state transition to $\chi_{i}^{c}\left(t+1\right)$, i.e., $a_{i}^{c}\left(t+1\right)≜\arg\max_{a_{i}^{c}\in A_{i}^{c}}Q^{c}\left(\chi_{i}^{c}\left(t+1\right),a_{i}^{c}|\omega_{t}\right)$. Then, the update to the DNN parameter $\omega_{t}$ is performed by the gradient descent method to minimize the mean square error between $Q_{i}^{c}\left(\chi_{i}^{c}\left(t\right),a_{i}^{c}\left(t\right)|\omega_{t}\right)$ and the target value $y_{i}^{c}\left(t\right)$ in (17), similar to (13). The size of the state and action spaces in each learning layer affects the computational complexity of the algorithm, which depends on the selection of parameters such as size, depth, learning rate, and discount factor. The deep neural networks of the lower-layer DQN method include three fully connected layers and two relu layers. The upper-layer MADDPG includes two sets of DNNs to approximate the Q network and the policy network, respectively. Each DNN in the MADDPG follows the same structure as that of the DQN method. The process is shown in Algorithm 2.
**Algorithm 2** Hierarchical learning for multi-UAV trajectory planning, transmission scheduling, and access control1:Initialize the observations of UAVs and GUs2:Collect state information $\left\{S^{o},S^{c}\right\}$3:%**Upper-layer MADDPG for trajectory learning**4:**for** each UAV i∈N **do**5:  Collect UAVs’ state information $s_{i}^{o}$6:     Execute the upper-layer action $a_{i}^{o}$7:     %**Lower-layer DQN for transmission learning**8:     Collect GUs’ state information $S_{i}^{c}$9:     Execute the lower-layer action $A_{i}^{c}$10:**end for**11:Update the joint action $\left\{A^{o},{\left[A_{i}^{c}\right]}_{i\in N}\right\}$12:Observe total reward function $\left\{R^{o},{\left[R_{i}^{c}\right]}_{i\in N}\right\}$13:Update all networks14:Loop back to Step (3)

## 6. Numerical Results

In this section, we evaluate the performance of the MADDPG and the hierarchical learning algorithms. Without loss of generality, we focus on a UAV-assisted wireless sensing network with one RBS and three UAVs assisting with information collection from a group of GUs randomly distributed in a two-dimensional coordinate system scaled to the range [−1,1]. All GUs are far away from the RBS and there are no direct links between the RBS and GUs. More-detailed parameters are listed in Table 1.

### 6.1. Convergence and Reward Performance

**Figure 4:**
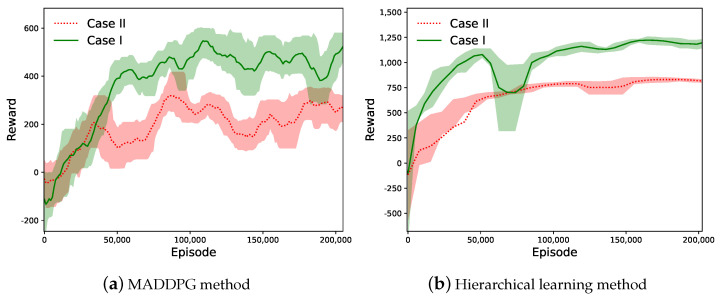
The reward dynamics of two algorithms for two cases.

Firstly, we evaluate the learning performance for UAV trajectory planning in the upper-layer learning phase considering two different cases. For Case I, we assume that all UAVs are initially deployed at random locations to serve the GUs. In Case II, the UAVs are assumed to take off from the same dispatch point. The reward dynamics in the conventional MADDPG and the proposed hierarchical learning method for the two cases are compared in Figure 4. By interacting with the environment and adapting UAV trajectories, the reward values in both methods increase and eventually stabilized after a number of iterations, which verifies the effectiveness and convergence of the proposed learning method. An interesting observation is that the reward in Case I is generally higher than that achieved in Case II. This implies that the UAVs’ initial dispatch locations are important to the overall sensing performance with the same data traffic distribution of the GUs. When the UAVs are scattered over the service coverage area, it can be faster and more efficient for the UAVs to find preferable sensing locations and trajectories to avoid service overlap and resource conflicts. When all UAVs start from the same location, there always exists some service overlap in the early stage of their trajectories. This implies inefficient cooperation among different UAVs and leads to reduced reward performance.

**Figure 5:**
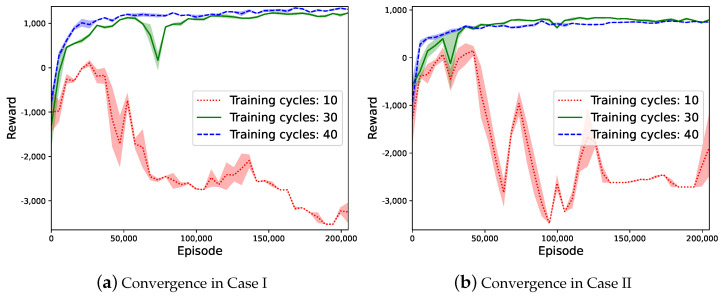
Performance comparison with different training cycles.

The convergence properties with different training cycles are shown in Figure 5. It reveals that a shorter training step has difficultly achieving convergence, as shown by the red dotted lines. The convergence results with training cycles of 30 and 40 are very similar. Hence, we set the training cycle to 30 in our simulations. We also test different combinations of learning parameters, including the learning rate, mini-batch size, replay buffer size, and discount factor, to help select the best hyperparameters for our experiments.

Compared with the conventional MADDPG, the reward curve of the hierarchical learning method is more stable and is smoother, as shown in Figure 4b. The hierarchical learning method achieves faster convergence and a much higher reward and stabilizes after 50 k learning episodes, while the MADDPG method still shows obvious fluctuations after 200 k learning episodes. A possible explanation of this observation is that the conventional MADDPG method adapts the high-dimensional control variables simultaneously, including UAV trajectory-planning and transmission-scheduling and GU access-control and mode-selection strategies, while the hierarchical learning method updates a smaller size of decision variables in each learning episode with reduced action space. Note that the UAVs’ space separation limits their interference with each other. As such, the hierarchical learning structure can avoid inefficient action combinations from the UAVs and the GUs, therefore reducing the overall action space and improving learning efficiency. Another advantage lies in that the hierarchical learning structure only requires the RBS to have limited communications with the UAVs. GU status information is not necessarily reported to the RBS for efficient trajectory planning and transmission scheduling. This avoids excessive communication overhead and latency in online learning.

### 6.2. Trajectory Planning in Two Cases

**Figure 6:**
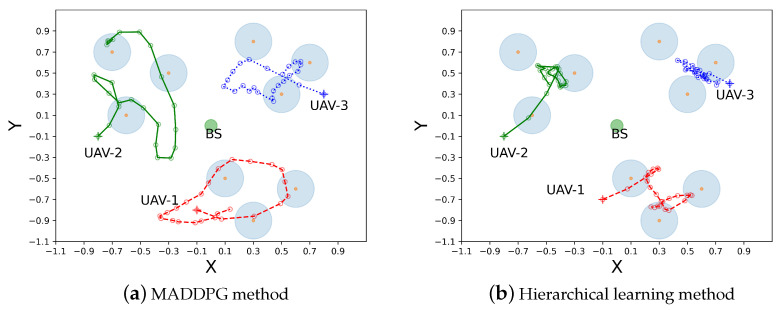
Case I: trajectory planning from different starting points.

**Figure 7:**
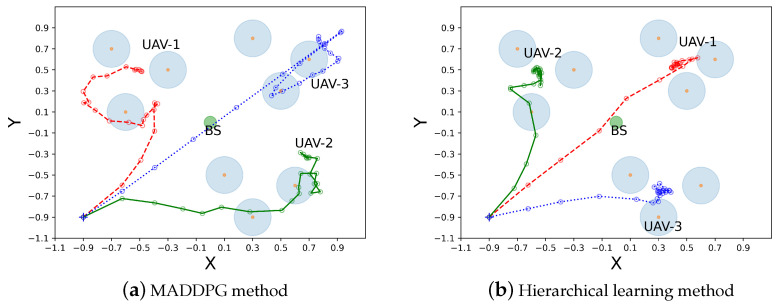
Case II: trajectory planning from the same starting point.

In Figure 6 and Figure 7, we compare UAV trajectory planning in 2D coordinate for two cases with different algorithms. The colored lines in these figures represent UAV trajectories, and the hollow circles represent UAV hovering points on the trajectories during different time slots. We observe that, after training, the UAVs can fly to different service regions without interfering with each other in both the MADDPG and the hierarchical learning algorithms. This shows the task collaboration of different UAVs to cover a large service area. As shown in Figure 6, though UAV trajectories are different with the two planning algorithms, each UAV intends to serve the closest group of GUs starting from the initial location. The hierarchical learning method can be more efficient, as UAV trajectories are confined to small service regions, as shown in Figure 6b, while UAV trajectories in the MADDPG method cover a larger area, as shown in Figure 6a. Similar observations are revealed in Case II, where all UAVs plan their trajectories from the same starting point. Both trajectory-planning algorithms ensure that the UAVs quickly reach their service regions to efficiently explore their task collaboration in a large-scale sensing network. For Case II, the trajectory comparison between two planning algorithms also verifies that the proposed hierarchical learning method achieves a more compact trajectory for each UAV compared to that of the conventional MADDPG method. This corroborates the more stable and faster learning performance shown in Figure 4. A possible explanation to this observation is that the conventional MADDPG needs to collect the status information from both the UAVs and GUs when making trajectory-planning decisions. The GUs’ random task arrivals and channel fluctuations may disturb UAV trajectories and thus create instability during the learning process. On the contrary, the hierarchical learning method only focuses on UAV status information and reduces the action space in the upper-layer trajectory planning. The GUs’ dynamic information is evaluated by individual UAVs and is used to assess the quality of the upper-layer trajectory planning.

### 6.3. Access Control and Buffer Dynamics

**Figure 8:**
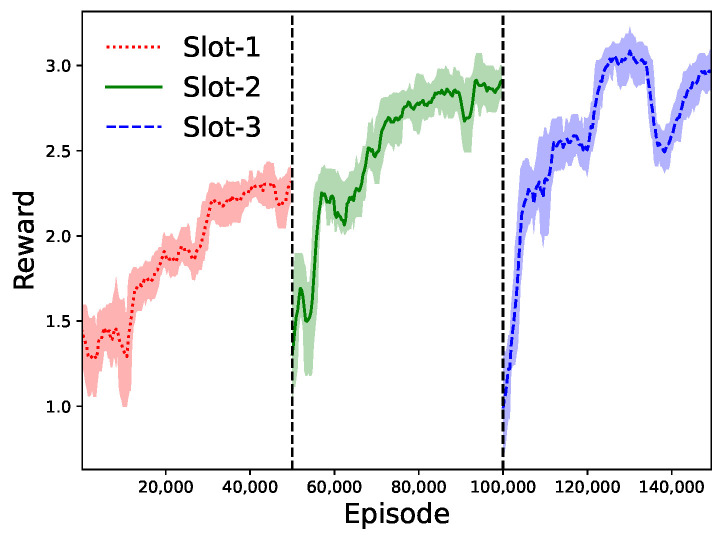
Reward dynamics in the lower-layer DQN algorithm.

In this part, we evaluate the GU access-control strategies in the hierarchical learning algorithm. Since the access-control strategy is updated by the lower-layer DQN method at each UAV, we can observe the dynamics of the reward function $R_{i}^{c}\left(t\right)$ in different time slots, as shown in Figure 8. Taking UAV-*i* as an example, we show the reward curves in three consecutive time slots for the UAV’s data sensing. During these time slots, the distances between UAV-*i* and the GUs are decreasing. It is clear that UAV-*i* can achieve a larger reward as it approaches the GUs gradually. One possible explanation is that both the GUs’ energy harvesting capabilities and the transmission rates for backscatter communications can be enhanced as the channel conditions between UAV-*i* and the GUs under its coverage improve. In each time slot, we can observe that UAV-*i*’s access-control strategy results in a gradually increasing reward function $R_{i}^{c}\left(t\right)$ until convergence after 30k learning episodes. Even if there is a performance drop in the reward curve, the UAV’s learning can quickly resume higher reward performance by adapting its access-control strategy, as shown in the third time slot in Figure 8.

**Figure 9:**
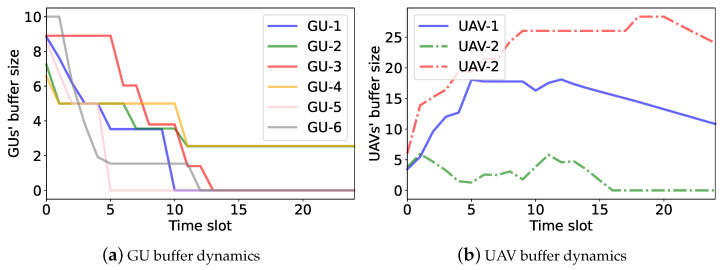
The buffer dynamics in the iDDPG algorithm.

**Figure 10:**
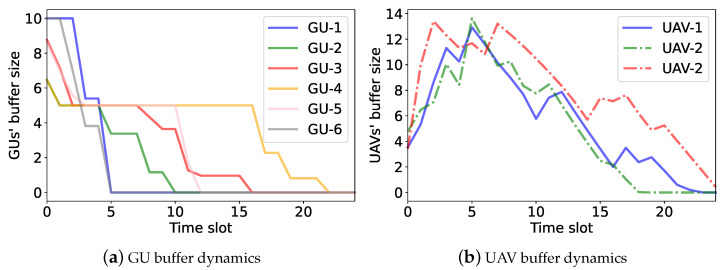
The buffer dynamics in the MADDPG algorithm.

**Figure 11:**
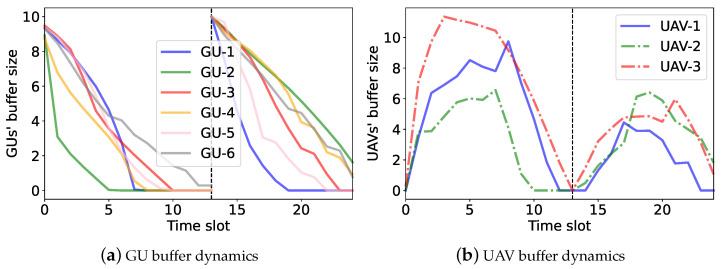
The buffer dynamics in the hierarchical learning algorithm.

We further verify the performance of the UAVs’ access-control strategies by examining the GUs’ and UAVs’ buffer dynamics, as shown in (4) and (5), respectively. A preferable access-control strategy ensures a stable buffer size and fairness among different GUs. For performance comparison, we introduce a single-agent independent DDPG (denoted as iDDPG) that regards each UAV as an independent agent. It allows each UAV to learn its own strategy independently based on its local observations. We apply iDDPG, MADDPG, and the proposed hierarchical learning algorithms to adapt the UAVs’ access-control strategies. The UAVs’ and the GUs’ buffer dynamics with different algorithms are shown in Figure 9, Figure 10 and Figure 11. In the simulation, we assume that all GUs constantly generate data traffic and the UAVs help forward GU data to the RBS. If the UAVs complete the data collection in advance, a new round of data collection can be carried out. The black dotted lines in Figure 10 and Figure 11 represent the beginning of a new round of data collection.

Figure 9 shows the dynamics of the GU and UAV buffer states over different time slots by the iDDPG algorithm. In Figure 9a, we can see that some GU data traffic cannot be completely collected by the UAVs and forwarded to the RBS in a timely manner. Hence, the buffer sizes drop slowly and still remain at non-negative values after 25 time slots. Additionally, as shown in Figure 9b, the UAV buffer sizes are unbalanced. This implies that the iDDPG algorithm cannot fully explore the UAVs’ task cooperation to maximize the overall energy efficiency. In the MADDPG algorithm, as shown in Figure 10, each UAV has its own service region and collects the GU data traffic more efficiently. By the UAVs’ cooperative operation, the GUs can deplete their data buffers faster and resume the next round of data collection, as shown in Figure 10a. Compared with the iDDPG algorithm, the UAVs’ data buffers are more balanced in the MADDPG algorithm. This is because the UAVs have different service regions and thus can avoid interfere with each other, as shown in Figure 10b. In the hierarchical learning algorithm, we find that the UAVs can complete data transmission faster, as their data buffers turn into zero and then start a new round of data collection, as shown in Figure 11. Moreover, the data collected by each UAV is well-balanced by the UAVs’ collaborative trajectory planning. This reveals that the hierarchical learning algorithm has higher energy efficiency compared to iDDPG and MADDPG.

**Figure 12:**
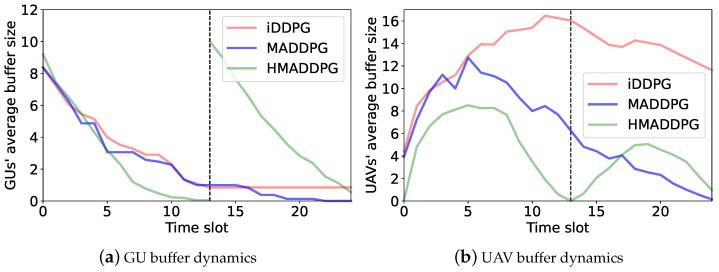
Average buffer size for three learning algorithms.

Figure 12 compares the GUs’ and the UAVs’ average buffer sizes in the iDDPG, MADDPG, and hierarchical learning (denoted as the HMADDPG) methods. The average buffer size slowly decreases in the iDDPG algorithm. The reason is that the UAVs cannot obtain all of the other UAVs’ status information when making trajectory-planning decisions, which may lead to suboptimal deployment locations for the UAVs and degrade the overall energy efficiency of the system. We also observe that the HMADDPG method achieves a faster decrease in the GUs’ average buffer size compared with the other methods, as shown in Figure 12a. The UAVs’ average buffer size also goes to zero at a much faster rate, as shown in Figure 12b. This implies that the HMADDPG method allows the UAVs to collect more GU sensing data compared to the other baselines by smartly adapting UAV trajectory and access-control strategies.

## 7. Conclusions and Future Work

In this paper, we proposed a hierarchical learning algorithm to maximize the sensing capacity of a multi-UAV-assisted sensing network by adapting the GUs’ access-control and mode-selection strategies as well as the UAVs’ transmission-scheduling and trajectory-planning strategies. Leveraging the distributed nature of the multi-UAV-assisted network, we proposed a hierarchical learning framework that decomposes the control variables into two layers. The upper-layer MADDPG algorithm is employed to adapt the UAV trajectory-planning and scheduling strategies based on UAV status information, while the lower-layer DQN algorithm is proposed to update the GU access-control and mode-selection strategies within each individual UAV’s service coverage area. Our numerical results show that the hierarchical learning algorithm can efficiently exploit UAV task cooperation and also improve overall learning efficiency. The distributed and hierarchical learning methods can improve data transmission performance in future UAV-assisted wireless networks. This allows UAVs to quickly adapt to the time-varying channel environment in a large-scale wireless network. However, practically, the hierarchical DRL learning scheme may still require a long time to train the lower-layer DQN for each upper-layer decision epoch. This results in an excessive run-time of the learning algorithm. In the future, we can consider improving the learning efficiency and accelerating the convergence speed of the lower-layer DQN method by integrating model-based local information.

## Figures and Tables

**Table 1:** Parameter settings in the numerical simulations.

Parameter	Setting
Training cycles per episode	30
Path-loss coefficient α	2
Range of GU’s data size $\zeta_{m}$	[5,15] Mbits
Maximum UAV speed $v_{\max}$	25 m/s
Noise power δ	−90 dBm
ϵ-greedy parameter	0.05
Actor’s learning rate	$10^{-3}$
Critic’s learning rate	$10^{-4}$
Batch size	32
Reward discount	0.95
Memory capacity	2000
Target replace iter	100

## Data Availability

Not applicable.

## Abbreviations

Unmanned Aerial Vehicle	UAV
Internet of Things	IoT
Ground User	GU
Remote Base Station	RBS
Block Coordinate Descent	BCD
Multi-agent Proximal Policy Optimization	MAPPO
Multi-agent Deep Deterministic Policy Gradient	MADDPG
Federated MADDPG	F-MADDPG
Federated Averaging	FA
Hierarchical Multi-Agent DRL	H-MADRL
Quality-of-Service	QoS
Mixed-Integer Nonlinear Programming	MINLP
Line-of-Sight	LOS
Markov Decision Process	MDP
Deep Neural Network	DNN
Independent DDPG	IDDPG
Non-Orthogonal Multiple Access	NOMA
Rate-Splitting Multiple Access	RSMA
